# Prioritizing Clinically Relevant Criteria for Longitudinal Obesity Management: A Systemic Framework to Support Decision-Making

**DOI:** 10.1007/s11695-026-08652-y

**Published:** 2026-04-16

**Authors:** Erica Luisa Correa, Rodrigo Strobel, Osiris Canciglieri Junior, Jones Luís Schaefer

**Affiliations:** 1 Industrial and Systems Engineering Graduate Program, Pontifícia Universidade Católica do Paraná, Curitiba, Brazil; 2 Our Lady Grace Hospital, Gastrovida - Bariatric and Metabolic Surgical, Curitiba, Brazil

**Keywords:** Obesity management, Obesity etiology, Clinical decision support, Treatment adherence, Multi-criteria decision making, Endocrine and metabolic mechanisms

## Abstract

**Introduction:**

Obesity is a multifactorial chronic condition characterised by the interplay of biological, behavioural, psychological, and environmental factors, as well as barriers that influence treatment adherence. This study aimed to identify, organise, and prioritise criteria for obesity management by proposing a systemic framework to support clinical decision-making.

**Methods:**

To this end, a systematic literature review was conducted to identify criteria related to obesity etiology, its consequences, barriers, and motivations for treatment. These criteria were then evaluated by an expert panel. Expert judgements were translated into linguistic scales within a fuzzy environment, and consensus was validated using the Fuzzy Delphi method. The Fuzzy TOPSIS method was then applied to calculate proximity coefficients and rank the criteria across two dimensions: importance and applicability. These dimensions were subsequently unified to generate a consolidated prioritisation for data collection.

**Results:**

The results highlight the centrality of dietary, emotional, and treatment-adherence factors, reinforcing the need to approach obesity from a multifactorial, non-reductionist perspective.

**Conclusion:**

The proposed framework supports planning clinical, surgical, and public health interventions, as well as the development of decision-support systems for longitudinal obesity management. Importantly, the framework does not diminish the relevance of endocrine and genetic mechanisms in obesity but prioritises criteria that are operationally feasible for routine clinical decision support systems, particularly in real-world and multidisciplinary obesity care settings.

## Key Points

• A systemic framework operationalizes obesity as a multifactorial condition.

• Adherence and continuity emerged as central drivers in obesity management.

• Clinically relevant obesity criteria were validated and prioritized by experts.

• A multicriteria approach structured and prioritized the obesity management criteria.

## Introduction

Obesity is a chronic, multifactorial, and rapidly growing condition, recognized as one of the major contemporary public health challenges [1]. Its etiology encompasses biological, behavioral, environmental, and psychological factors whose complex interactions result not only in excessive body fat accumulation but also in a broad spectrum of obesity-related diseases (ORDs), including type 2 diabetes, cardiovascular diseases, dyslipidemia, nonalcoholic steatohepatitis, and certain types of cancer [2]. The strong association between obesity and these comorbidities reinforces its systemic nature and underscores its substantial impact on patient morbidity and mortality [2]. Beyond its clinical consequences, obesity also has profound social and psychological repercussions, directly affecting the quality of life of individuals living with the condition [3, 4]. This complexity demands management strategies that move beyond reductionist approaches. Such approaches typically address obesity in a simplified manner, focusing exclusively on weight control through diet and physical activity, while neglecting the psychological, behavioral, environmental, and cultural factors that influence its development and persistence.

These limited perspectives tend to place responsibility solely on the individual, overlooking the multifactorial nature of obesity [5], thereby contributing to stigma and poor treatment adherence [6]. As a result, this restricted view may inadvertently favor the persistence and progression of the condition.

In this context, there is a clear need for a more comprehensive management model that integrates multiple dimensions of care, ranging from prevention and in-depth diagnosis to personalized treatment strategies [7]. Such a model requires a clear, well-structured definition of clinical and contextual criteria to guide decision-making at each stage of care, thereby enabling more precise and effective interventions.

To address this need, the present study adopts a structured approach organized into three interdependent phases: prevention, diagnosis, and treatment. This approach was informed by the World Health Organization (WHO) Preparedness, Readiness and Response (PRET) framework, originally developed for respiratory and infectious diseases [8]. Through critical analysis and adaptation, this framework was reinterpreted for obesity, a chronic noncommunicable condition with epidemic-like patterns of prevalence and impact, while preserving the structural logic of PRET and incorporating elements specific to its multifactorial complexity.

Within this framework, emerging technologies such as artificial intelligence (AI) have the potential to enhance analytical capacity and clinical decision-making by linking large volumes of data to relevant clinical patterns and supporting management across all phases of care (prevention, diagnosis, and treatment) [9]. For such technological applications to be effective, however, robust, well-defined, and prioritized criteria are required to serve as reliable inputs for the development and operation of decision-support algorithms.

Against this background, the central objective of this study is to develop a multifactorial framework for obesity management that integrates AI-based decision support with Multi-Criteria Decision-Making (MCDM) methods grounded in fuzzy logic. Specifically, the Fuzzy Delphi Method (FDM) and the Fuzzy Technique for Order Preference by Similarity to Ideal Solution (TOPSIS) are used to validate, weight, and rank criteria related to obesity etiology, consequences, and barriers to treatment, as identified through systematic literature reviews and expert consensus. The expected contributions of this study are threefold. At the theoretical level, it proposes a conceptual model that frames obesity as a multifactorial system, positioning etiology at its core and elucidating its relationships with consequential and treatment-related factors. At the practical level, the framework provides a technical and operational reference for determining which data should be collected to support the development of AI-based models and decision-support tools. Finally, at the social level, the proposed framework aims to deepen understanding of obesity as a complex condition, contribute to stigma reduction, inform policy, and promote more equitable management approaches, including the responsible integration of emerging technologies into real-world care settings.

### PRET Initiative Adapted for Obesity Management

Although obesity is a chronic noncommunicable condition, it exhibits epidemic-like dynamics in terms of prevalence and impact [10]. Considering this characteristic, the present study draws on the structural logic of the PRET framework, originally organized around prevention, preparedness, response, and resilience, and adapts it to the context of obesity management. Rather than transferring the PRET model directly, its core principles were reinterpreted to inform a phase-based approach to obesity care, organized into three interdependent phases: prevention, diagnosis, and treatment.

In the prevention phase, the objective is to reduce the incidence of new cases through population-based and individual-level strategies that modify risk factors and promote healthy lifestyle behaviors [11]. Effective prevention not only limits the growth of obesity prevalence but also alleviates downstream pressure on diagnostic and treatment stages. At this phase, AI may support the monitoring of population trends, early identification of at-risk groups, detection of relevant predictors, and personalization of preventive recommendations based on behavioral, environmental, and clinical data.

The diagnosis phase extends the traditional approach, which is often restricted to obesity severity classification (grades I, II, and III), by incorporating a more in-depth assessment of underlying causes and consequences within a systemic perspective [12]. This broader diagnostic view enables the joint management of obesity and its comorbidities while accounting for prior treatment history and individual patient responses. In this context, AI can assist in integrating heterogeneous clinical and contextual data, in identifying complex patterns that are not readily apparent through conventional analyses, and in supporting risk stratification and therapeutic prioritization.

The treatment phase encompasses not only clinical and behavioral interventions but also the identification and management of barriers to both treatment initiation and continuity. Barriers to initiation refer to factors that prevent patients from engaging in care [13], whereas barriers to continuity are associated with treatment interruption or abandonment [14]. Addressing these barriers alongside an expanded diagnostic assessment facilitates the development of more individualized and sustainable treatment strategies. At this stage, AI may support the selection of the most appropriate therapeutic approach for different patient profiles, predict the risk of treatment discontinuation based on clinical and contextual factors, and enable real-time monitoring of adherence and the adjustment of adaptive strategies.

By structuring obesity management into these three phases, the proposed model provides a coherent framework for addressing obesity’s multifactorial nature and lays the groundwork for integrating emerging technologies. In this context, defining robust, hierarchically organized clinical and contextual criteria is a critical step. Accordingly, applying multicriteria methods grounded in fuzzy logic to validate, weight, and rank criteria related to etiology, consequences, and treatment barriers supports both the refinement of clinical practice and the development of AI-enabled decision-support tools applicable across diverse obesity care settings.

## Materials and Methods

**Fig. 1:**
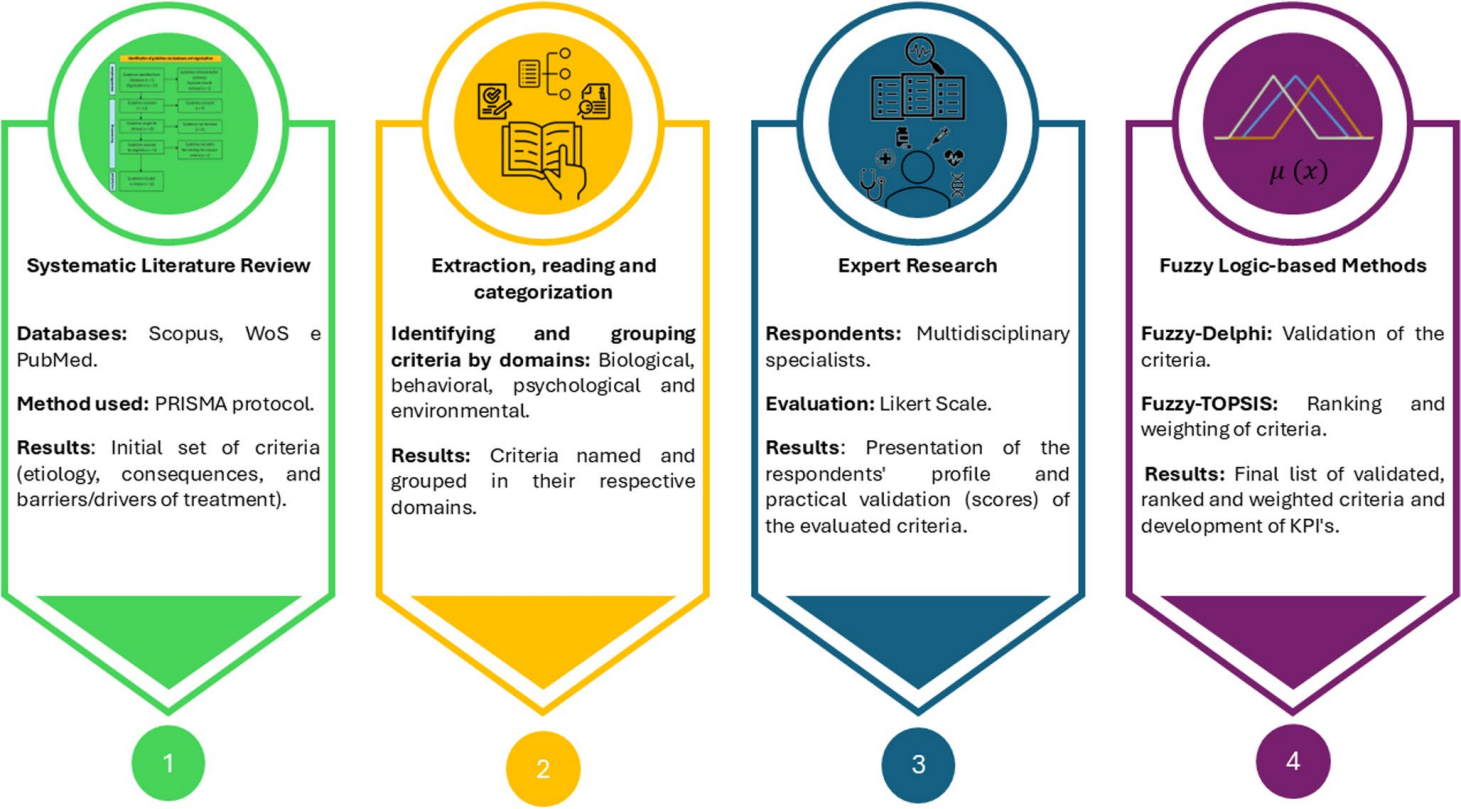
Research stages

This study adopted an applied, descriptive, and exploratory design, integrating qualitative and quantitative approaches to develop a clinically grounded framework for obesity management. The methodological strategy was structured into four sequential stages, designed to ensure transparency, reproducibility, and clinical relevance. These stages comprised: (i) a Systematic Literature Review (SLR) to identify clinically meaningful criteria related to obesity etiology, consequences, and treatment adherence; (ii) extraction, reading, and categorization to synthetize these criteria; (iii) validation and assessment of criteria by a multidisciplinary expert panel; and (iv) prioritization and weighting of criteria using fuzzy logic-based MCDM methods. Figure 1 summarizes the methodological workflow and corresponding outputs.

### First Stage – Systematic Literature Review

The first stage consisted of a qualitative systematic literature review conducted in accordance with the PRISMA (Preferred Reporting Items for Systematic Reviews and Meta-Analyses) 2020 guidelines, ensuring transparency and reproducibility in study selection [15]. The objective of this review was to identify criteria reflecting the multifactorial etiology and complexity of obesity, including etiological factors, clinical consequences, and barriers and motivators related to treatment initiation, adherence, and discontinuation.

Searches were performed in the Scopus, Web of Science, and PubMed databases, including peer-reviewed articles without time restrictions. Two thematic axes guided the search strategy: (i) etiology and consequences of obesity and (ii) barriers and motivators related to obesity treatment. Search strings were adapted to each database and are detailed in Table 1. Records were screened using Zotero reference management software to remove duplicates and retracted articles [16].

**Table 1:** Search strategy

Thematic axis I		Thematic axis II	
Pillar	Keywords	Pillar	Keywords
Obesity	Obesity; Overweight	Obesity	Obesity; Overweight
Obesity Consequences	Obesity-related diseases; Psychological consequences; Physical consequences; Socio-environmental consequences; Socioeconomic consequences; Environmental consequences; Economic consequences; Social consequences	Obesity Treatments	Obesity treatments; Obesity intervention; Adherence to treatment; Motivation for treatment; Barriers to treatment; Treatment complications; Stages of obesity treatment; Success factors in treatment
Obesity Etiology	Etiology; Causes; Psychological causes; Physical causes, Socio-environmental causes; Socioeconomic causes; Environmental causes; Economic causes; Social causes; Psychological factors; Physical factors; Socio-environmental factors; Socioeconomic factors; Environmental factors; Economic factors; Social factors	Quality of life	Quality of life; Evaluation of quality of life; physical and emotional well-being; Health-related quality of life; Quality of life assessment criteria
Search results			
Databases	Scopus	WoS	PubMed
No Filter	**4536**	**2689**	**2643**
With Filter	English and Article	English and Article	English and Human
	**2698**	**1865**	**1737**

The systematic search conducted in August 2025 yielded 6,309 records across the three databases. After removal of duplicates (*n* = 2,668) and exclusion of four retracted articles, 3,637 records remained for title and abstract screening. At this stage, studies were assessed according to the predefined thematic axes. Records that investigated the etiology of diseases other than obesity, or that referred to the “etiology of obesity” only in a contextual manner without treating it as a central focus, were excluded. Likewise, studies in which obesity appeared solely as a secondary characteristic, risk factor, or predictor of other conditions were removed. In contrast, studies addressing difficulties patients face during treatment, as well as criteria related to engagement, adherence, and continuity of care, were retained because they aligned with the scope of the review.

Following this screening process, 568 records remained in thematic axis I (etiology and consequences of obesity), and 188 records remained in thematic axis II (barriers and motivators of obesity treatment). Based on these subsets, specific inclusion and exclusion criteria were defined for each thematic axis to guide the subsequent eligibility assessment.

For thematic axis I, inclusion criteria required that studies empirically investigate or demonstrate causal or contributory relationships between psychological, behavioral, biological, or environmental factors and the development of obesity, or analyze the consequences of obesity with explicit linkage to these domains. Applying these criteria reduced the dataset from 568 to 231 records. For thematic axis II, inclusion criteria required that studies investigate factors influencing decisions to initiate, maintain, or discontinue obesity treatment and explicitly address barriers and/or facilitators of adherence. Applying these criteria reduced the set from 188 to 104 records.

The eligibility assessment was then conducted using predefined exclusion criteria. For thematic axis I, studies were excluded if they: (i) did not focus on factors classifiable as psychological, behavioral, biological, or environmental, addressing obesity only in a general or descriptive manner without explicit mechanisms; (ii) were based solely on opinions or speculative arguments; or (iii) had substantial methodological limitations or focused on other diseases as the primary subject. For thematic axis II, studies were excluded if they: (i) focused exclusively on the effectiveness of weight loss interventions without addressing adherence-related factors; (ii) examined populations in which obesity was not the primary condition (e.g., terminal illness or eating disorders unrelated to obesity); or (iii) failed to provide evidence or insights into reasons for treatment initiation, maintenance, or discontinuation, including relevant barriers to adherence.

**Fig. 2:**
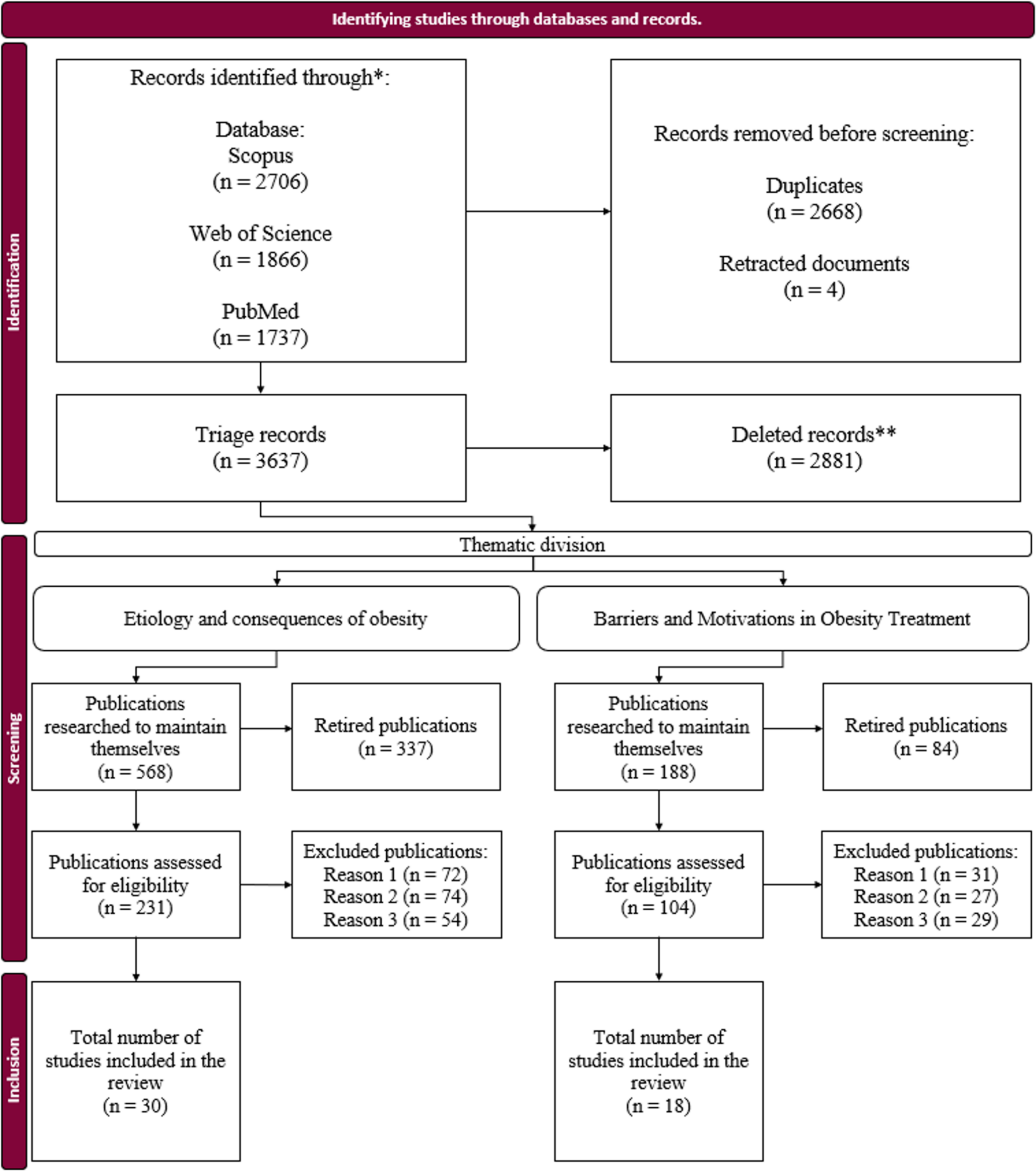
PRISMA flowchart

After systematically applying these criteria, the final sample comprised 30 studies addressing the etiological factors and consequences of obesity and 18 studies focusing on barriers and drivers of treatment. Data extraction was conducted objectively and limited to criteria related to the causes, consequences, barriers, and motivators associated with obesity management. The number of records retained at each stage and the overall selection process are summarized in the PRISMA flow diagram in Fig. 2.

### Second Stage – Extraction, Reading and Categorization

The second stage aimed to transform the evidence identified in the SLR into a set of clinically meaningful and operational criteria to support obesity management across phases of care. In this stage, data extraction focused on identifying factors related to obesity etiology, its consequences, and barriers and motivators associated with treatment initiation, adherence, and continuity.

For the etiology axis, all factors described in the selected studies as causes, determinants, predictors, or risk factors for the onset or progression of obesity were coded as criteria, spanning biological, behavioral, psychological, and environmental dimensions. For the consequences axis, elements characterizing physical, psychological, social, or economic losses were extracted when presented as resulting from or strongly associated with obesity, including obesity-related diseases, emotional distress, stigma, and functional limitations. For the barriers and motivators axis, factors influencing decisions to initiate treatment, maintain adherence over time, or discontinue care were identified, including reasons reported by patients and authors’ interpretations of facilitators and impediments, such as social support, financial conditions, access to services, and experiences of prejudice.

All relevant excerpts were compiled and subjected to an iterative synthesis process. Initially, an extensive list of factors was generated, including semantic overlaps and redundancies. These factors were then grouped into broader categories within shared conceptual domains while preserving their original clinical meaning. For example, specific references to “fatty foods,” “sugary drinks,” “fast food,” or “ultra-processed foods” were consolidated into a broader criterion reflecting an unbalanced diet characterized by hyperpalatable or ultra-processed food consumption. A similar aggregation process was applied across the remaining dimensions.

Some factors were intentionally retained in more than one category when empirical evidence supported that classification. For instance, family and social support were identified as motivators for treatment initiation and maintenance, and as barriers when absent. This approach reflects the multifactorial and interconnected nature of obesity-related determinants and aligns with clinical practice, in which the same factor may play distinct roles at different stages of care.

This stage was complemented by structured discussions among the authors, which refined the clinical perspective adopted in the study and guided the identification of potentially relevant information. These discussions supported conceptual alignment and ensured that the synthesized criteria remained clinically interpretable and applicable across diverse obesity care settings.

At the conclusion of this stage, a consolidated and structured set of criteria was obtained, organized into etiological factors, consequences of obesity, and barriers and motivators related to treatment. This final set of criteria served as the input for the subsequent expert validation and multicriteria analysis stages.

### Third Stage – Expert Research

In the third stage, the criteria identified and synthesized in the previous stages were evaluated by a multidisciplinary panel of 25 specialists actively involved in obesity management. The panel included professionals from eight strategic areas: bariatric and metabolic surgery (*n* = 6), endocrinology (*n* = 4), nutrition (*n* = 4), psychology (*n* = 4), nursing (*n* = 3), cardiology (*n* = 2), physiotherapy (*n* = 1), and physical education (*n* = 1).

Although multidisciplinary teams are formally recommended in bariatric surgery, this same logic was deliberately extended to obesity management as a whole. This decision reflects the understanding that obesity, regardless of therapeutic pathway, requires an integrated approach encompassing the metabolic, behavioral, psychological, functional, and psychosocial dimensions of care [17]. Accordingly, the composition of the expert panel was designed to capture complementary clinical perspectives relevant to the complexity of obesity management.

With respect to professional experience, eight participants reported 0–5 years of practice, four reported 6–10 years, four reported 11–20 years, and nine reported more than 20 years of experience. Most participants held postgraduate qualifications, predominantly specialization degrees, with a smaller number holding master’s and doctoral degrees. Levels of professional involvement in obesity management were predominantly high: nine experts identified as specialists in the field, nine reported high involvement, six moderate involvement, and one low involvement. Clinical exposure was also substantial, with nearly half of the panel reporting daily care of patients with obesity.

Data collection was conducted using an online questionnaire administered via Google Forms, chosen for its accessibility and device compatibility, thereby facilitating expert participation [18]. The questionnaire was structured into four sections: (i) presentation of the study objectives and informed consent, including information on data use in accordance with the Brazilian General Data Protection Law (LGPD), Law No. 13,709/2018) [19], (ii) respondent profile, used to characterize expertise and verify internal consistency, (iii) evaluation of the criteria identified in the SLR, and (iv) an open-ended field for comments and qualitative input.

Criteria were evaluated using a five-point Likert scale across two dimensions: importance (clinical relevance) and applicability (feasibility of routine data collection in clinical practice). Etiological criteria (C1-C20) were evaluated individually, reflecting their central role in the proposed framework. In contrast, criteria related to consequences and to barriers and motivators of treatment were evaluated as aggregated aspects. Specifically, consequences were grouped into biological (C21), psychological (C22), and behavioral (C23) aspects, while treatment-related factors were grouped as motivation for treatment initiation (C24), barriers to initiation (C25), motivation for treatment discontinuation (C26), and motivation for treatment continuity (C27). This aggregation strategy was adopted to balance methodological rigor with feasibility, reducing questionnaire length, minimizing respondent fatigue, and lowering the risk of survey abandonment, while preserving the clinical interpretability of the evaluated constructs. The resulting dataset provided the basis for the subsequent fuzzy Delphi validation and fuzzy TOPSIS prioritization stages.

### Fourth Stage – Fuzz Logic-Based Methods

In the fourth stage, fuzzy logic-based MCDM methods were applied to validate, weight, and prioritize the criteria evaluated by the expert panel. Specifically, the Fuzzy Delphi Method (FDM) was first used to assess consensus and support the validation of criteria under conditions of uncertainty, subjectivity, and linguistic judgment, as widely reported in health-related decision analysis studies [20–22].

Expert evaluations were initially collected using a five-point Likert scale and subsequently converted to triangular fuzzy numbers (TFNs), allowing uncertainty and variability in clinical judgment to be explicitly represented. The linguistic terms and fuzzy scales used for relevance and applicability are listed in Table 2.

**Table 2:** Linguistic terms and fuzzy scales used

Likert scale	Linguistics terms for relevance	Triangular fuzzy numbers	Escala Likert	Linguistic terms for aplicabillity	Triangular fuzzy numbers
1	Not very relevant	(1, 1, 1)	1	Very difficult to obtain	(1, 1, 1)
2	Reasonably relevant	(1, 3, 5)	2	Difficult to obtain	(1, 3, 5)
3	Moderately relevant	(3, 5, 7)	3	Moderately accessible	(3, 5, 7)
4	Very relevant	(5, 7, 9)	4	Easily accessible	(5, 7, 9)
5	Extremely relevant	(7, 9, 9)	5	Completely accessible	(7, 9, 9)

The mapping of linguistic terms to TFNs is formalized in Eq. (1), which associates each ordinal response with the minimum, geometric mean, and maximum values of the experts’ evaluations $\:\left({L}_{i},\:{M}_{i},\:{U}_{i}\right)$. This transformation avoids premature loss of information that would occur if expert perceptions were reduced directly to single deterministic scores.

To represent each criterion with a single, interpretable value suitable for comparison, the aggregated TFNs were defuzzified using the center-of-gravity method, a commonly adopted procedure in fuzzy decision-making applications [23]. The defuzzification process converts the uncertainty embedded in expert evaluations into a scalar value, facilitating subsequent analysis while preserving the relative influence of expert consensus. Equation (1) presents the defuzzification.

1 $$\:{G}_{i}=\:\left(\frac{\left({U}_{i}-\:{L}_{i}\right)+({M}_{i}-\:{L}_{i})}{3}\right)+\:{L}_{i}$$

After obtaining a defuzzified value $\:{G}_{i}$ for each criterion, a cut-off threshold $\:{\upalpha\:}$ was defined to represent the minimum level of relevance and agreement expected within the FDM context. The threshold value was derived from the distribution of the $\:{G}_{i}$ values, and only criteria with $\:{G}_{i}>{\upalpha\:}$ were retained for subsequent analysis, as they met the minimum significance requirements for relevance and expert consensus [23]. This filtering step reduces the influence of less relevant criteria and ensures that the subsequent weighting and ranking stages focus on the most clinically meaningful factors.

After validating the criteria through the FDM, the fuzzy TOPSIS method was applied to weight and rank the criteria based on their relative importance and applicability. This method was chosen for its ability to handle multiple evaluation dimensions simultaneously and to manage uncertainty inherent in expert-based assessments, which is particularly relevant in complex clinical decision-making contexts [24].

Initially, a fuzzy decision matrix $\:\stackrel{\sim}{D}\:={\left[{\stackrel{\sim}{x}}_{ij}\right]}_{m\:\times\:\:n}$ was constructed, where each $\:{\stackrel{\sim}{x}}_{ij}$ is a TFN representing the aggregated $\:\:\left({l}_{ij},\:{m}_{ij},\:{u}_{ij}\right)$ expert evaluations for each criterion [24]. The decision matrix was normalized using a linear transformation, described as $\:\stackrel{\sim}{R}=\:{\left[{\stackrel{\sim}{r}}_{ij}\right]}_{m\:\times\:\:n}$. As all criteria evaluated in this study were of a benefit nature, higher values consistently indicate greater relevance or feasibility for obesity management. Therefore, a benefit-type normalization procedure was applied to the fuzzy decision matrix, ensuring comparability among criteria while preserving their relative magnitudes. No cost-type criteria were considered, as all evaluated factors contribute positively when present. To obtain the normalized matrix, Eq. (2) was applied.

2 $$\:{\stackrel{\sim}{r}}_{ij}=\:\left(\frac{{l}_{ij}}{{u}_{j}^{+}},\:\frac{{m}_{ij}}{{u}_{j}^{+}},\:\frac{{u}_{ij}}{{u}_{j}^{+}}\right),\:{u}_{j}^{+}=\:\begin{array}{c}max\\\:i\end{array}\:{u}_{ij}$$

Subsequently, the weighted matrix was obtained $\:\stackrel{\sim}{V}=\:{\left[{\stackrel{\sim}{v}}_{ij}\right]}_{m\:\times\:\:n}$, where the elements $\:{\stackrel{\sim}{r}}_{ij}$ of the normalized matrix$\:\:(\stackrel{\sim}{R)}$ are multiplied by a weight vector $\:\stackrel{\sim}{W}$ also expressed with TFN $\:\left({w}_{j}^{L},\:{w}_{j}^{M},\:{w}_{j}^{U}\right)$, and the weight vector must necessarily meet the rule $\:\sum\:_{j=1}^{n}{\stackrel{\sim}{w}}_{j}=1\:$ [24]. As a result, if the weight vector is not normalized, normalization must be performed component-wise, preserving the TFN’s uncertainty. Equation (3) shows this procedure.

3 $$\:{w}_{j}^{{L}^{{\prime\:}}}=\frac{{w}_{j}^{L}}{\sum\:_{j=1}^{n}{w}_{j}^{L}};{w}_{j}^{{M}^{{\prime\:}}}=\frac{{w}_{j}^{M}}{\sum\:_{j=1}^{n}{w}_{j}^{M}};{w}_{j}^{{L}^{{\prime\:}}}=\frac{{w}_{j}^{U}}{\sum\:_{j=1}^{n}{w}_{j}^{U}}$$

With the weighted matrix, $\:\stackrel{\sim}{V}j$ the positive ideal solution (FPIS) $\:{A}^{+}$ and the negative solution (FNIS) $\:{A}^{-}$ were defined for each criterion. Then, the distances of each alternative $\:i$ to these ideals were calculated by the vertex method [24]. Equations (4) and (5) shows these procedures.

4 $$\:{d}_{v}\left(\stackrel{\sim}{x},\:\stackrel{\sim}{z}\right)=\:\sqrt{\frac{1}{3}\left[{\left({l}_{x}-\:{l}_{z}\right)}^{2}+\:{\left({m}_{x}-\:{m}_{z}\right)}^{2}+\:{\left({u}_{x}-\:{u}_{z}\right)}^{2}\right]}$$

5 $$\:{D}_{i}^{+}=\:{\sum\:}_{j=1}^{n}{d}_{v}({\stackrel{\sim}{v}}_{ij},\:{\stackrel{\sim}{v}}_{j}^{+});\:\:{D}_{i}^{-}=\:{\sum\:}_{j=1}^{n}{d}_{v}({\stackrel{\sim}{v}}_{ij},\:{\stackrel{\sim}{v}}_{j}^{-})$$

Finally, the approximation coefficient $\:{CC}_{i}$ for each of the multifactorial and complex obesity criteria evaluated by the experts was calculated [24] (Eq. (6)).

6 $$\:{CC}_{i}=\:\frac{{D}_{i}^{-}}{\left({D}_{i}^{+}+\:{D}_{i}^{-}\right)}$$

In short, the FDM results were used to reach consensus and refine the initial list of criteria. The fuzzy TOPSIS method was applied separately to the importance and applicability dimensions, yielding two distinct rankings. These rankings were subsequently integrated to support decision-making, acknowledging that, in real-world clinical contexts, relevance must be balanced against the feasibility of data collection and implementation. The resulting unified ranking was used to guide the prioritization of criteria within the proposed framework.

## Results

This section presents the results of the SLR, beginning with the identification of criteria related to the etiology and consequences of obesity, followed by the presentation of barriers and motivators associated with obesity treatment. Subsequently, the proposed framework for obesity management is presented.

### Etiology and Consequences of Obesity

Within thematic axis I, a total of 30 studies were identified. Analysis of these studies revealed four integrated domains underlying obesity etiology: biological, behavioral, psychological, and environmental.

In the biological domain, studies by Foraita et al. [25], Mohammed et al. [26], Park e Choi [27], Prodan et al. [28], Sohn [29], Ludwig et al. [30], and Archer e Lavie [31] primarily examined genetic and pathophysiological mechanisms. These investigations examined genetic variants, obesity subtypes, and metabolic and hormonal alterations, underscoring the relevance of precision medicine and personalized strategies for identifying high-risk groups. Complementarily, Sertie et al. [32], Mihaylova et al. [33], and Da Fonseca et al. [34] emphasized gene-environment interactions and life-course factors, including intrauterine nutritional stress and specific molecular pathways, supporting the importance of early interventions and the consideration of individual susceptibility.

The behavioral domain was highlighted in studies by Jessri et al. [35], Worsley et al. [36], Brunner et al. [37], Lustig et al. [38], Martinez [39], and Jebb [40]. These works examined dietary patterns characterized by high energy density, irregular meal timing, and increased consumption of foods of low nutritional quality, linking these behaviors to na elevated risk of weight gain and obesity.

In parallel, the environmental domain emerged when these same authors, as well as Lemamsha et al. [41], associated dietary choices and physical activity levels with living conditions and daily-life structures, such as food availability, work environments, social organization, and urban design. These findings indicate that obesity etiology cannot be reduced to individual behavior alone but is embedded in broader contextual and structural determinants.

Finally, the psychological domain was supported by studies examining psychosocial and emotional determinants of the onset and maintenance of excess weight. Gonçalves et al. [42] explored perceptions among adolescents and families, identifying factors such as anxiety, depression, and stressful life events, including divorce and bereavement, combined with emotional eating as a coping strategy, alongside body shame and low self-esteem Brunner et al. [37] reinforced this domain by demonstrating, in a prospective design, that chronic occupational stress is associated with obesity development, partially mediated by behavioral pathways and neuroendocrine mechanisms. Additionally, Lustig et al. [38] highlighted psychosocial consequences of obesity, including depression and social isolation, contributing to the spectrum of relevant psychological outcomes.

Based on this synthesis, etiological criteria were organized into the four domains described above. In contrast, criteria related to obesity’s consequences were grouped into three domains, namely biological, psychological, and behavioral, because obesity primarily produces downstream effects at the individual level rather than constituting an environmental consequence per se. Importantly, behavioral determinants and weight-related outcomes operate through multiple pathways, involving bidirectional interactions and potential feedback loops [43]. Among the most frequently reported etiological criteria were diets rich in ultra-processed foods, sedentary behavior, high sugar consumption, genetic predisposition, and hormonal imbalances. Table 3 summarizes the etiological and consequential criteria identified in thematic axis I, along with their respective references, forming the basis for subsequent expert validation and multicriteria prioritization.

**Table 3:** Etiology and consequences of obesity criteria

Aspects	Code	Etiology criteria	References
Behavioral	C1	Irregular meal times (irregular schedules, irregular patterns)	[40, 41, 44–46]
	C2	Unbalanced diet and/or diet based on ultra-processed and hyperpalatable foods	[30, 31, 34–36, 39–42, 44–51]
	C3	Excessive consumption of sugar and sugary drinks	[30, 35, 36, 41, 44, 45, 47, 49, 51]
	C4	Excessive alcohol consumption	[35–37, 42, 46]
	C5	Sedentary lifestyle (low physical activity)	[30, 31, 34–37, 39–42, 45–52]
	C6	Sleep cycle (sleep deprivation, circadian dysregulation)	[33, 34, 41, 53]
Psychological	C7	Chronic and/or occupational stress affecting eating habits	[35, 37, 42, 44, 46]
	C8	Anxiety and emotional disorders	[42, 48]
	C9	Low self-esteem related to body image	[34, 42, 48]
	C10	Emotional eating (eating as a response to negative emotional states)	[42, 48, 54]
Biological	C11	Genetic predisposition, rare genetic obesity and polymorphisms (genes such as LEP, FTO, MC4R and GHRL)	[26–30, 33, 34, 36, 39, 40, 48, 50, 52–54]
	C12	Hormonal imbalances (leptin resistance, ghrelin alterations, insulin resistance/hyperinsulinemia, defect in hunger/satiety regulation in the hypothalamus)	[25, 26, 28–34, 37–40, 48, 50, 52, 54]
	C13	Endocrine diseases (hypothyroidism, Cushing’s syndrome and other endocrine conditions)	[34, 38, 48, 53]
	C14	Metabolic alterations (Reduction in basal energy expenditure)	[33, 34, 39, 40, 48, 50, 54]
	C15	Epigenetics, intrauterine programming and/or influence of the gut microbiota on the regulation of metabolism and predisposition to obesity	[32, 52, 55]
Environmental	C16	Obesogenic environment (excess of available high-calorie foods, lack of spaces for exercise)	[30, 36, 39–42, 44, 45, 47, 49, 50, 52]
	C17	Family influence and cultural norms	[25, 36, 39, 41, 42, 44, 47, 49, 52, 55]
	C18	Exposure to endocrine disruptors (phthalates and Bisphenol A)	[30, 34, 53]
	C19	Socioeconomic inequality affecting access to healthy foods and physical activity among different socioeconomic groups.	[25, 35, 40, 41, 45, 47, 49, 50]
	C20	Urban infrastructure, school environment and public policies as risk factors for obesity	[35, 36, 39–41, 45, 47, 49, 51]
Aspects	Number	Consequences criteria	References
Biological (Code - C21)	1	Cardiovascular diseases (hypertension, heart failure)	[26, 31, 34, 35, 37, 38, 46, 48, 50, 51, 54]
	2	Type 2 diabetes and insulin resistance	[26, 28–31, 33–35, 37, 38, 40, 48, 50–54]
	3	Metabolic disorders and chronic inflammation	[31, 33, 34, 38, 47, 48, 50, 54]
	4	Hormonal changes (such as hypothyroidism, Cushing’s syndrome)	[34, 48]
	5	Changes in renal and hepatic function (non-alcoholic fatty liver disease and nephropathies)	[26, 29, 34, 48, 50]
	6	Increased risk of premature mortality	[34, 37, 41, 46, 48, 50, 54]
	7	Effects on fertility (hormonal changes impacting fertility, especially in women due to ovarian dysfunction)	[29, 34, 50, 54]
Psycological (Code - C22)	8	Depression and anxiety associated with stigma and body image	[34, 38, 41]
	9	Low self-esteem exacerbating mental health problems	[34, 42]
	10	Social stigma contributing to isolation and emotional problems	[38, 42, 47]
Behavioral (Code - C23)	11	Reduced mobility and willingness to exercise, exacerbating sedentary lifestyles	[42, 47]
	12	Metabolic/hormonal changes and epigenetic and satiety mechanisms may contribute to hyperphagia cycles	[31, 34, 48, 54]

### Barriers and Treatment Motivators

Within thematic axis II, a total of 18 records were included. Analysis of these studies revealed four integrated dimensions related to treatment-seeking behavior, adherence, and continuity in obesity care: motivators for treatment initiation, barriers to initiation, barriers to continuity (discontinuation), and motivators for continuity.

With respect to motivators for treatment initiation, population-based studies focusing on health perception, such as those by Salle et al. [56] and Look et al. [57], indicate that the decision to seek treatment is commonly triggered by expectations of improved health and well-being, as well as by the perceived need for more structured guidance within the care pathway. These findings reinforce the importance of explicitly addressing obesity in clinical encounters and ensuring clarity about therapeutic options and management trajectories. In clinical and family-based interventions, motivation to initiate treatment is further enhanced when engagement strategies are implemented from the first contact. Communication approaches that reduce resistance and increase acceptance of follow-up care have been shown to facilitate treatment entry, particularly in pediatric and family-centered programs that incorporate active support and care organization to promote initial attendance, as reported by Taveras et al. [58] and Bean et al. [59]. In specific populations, such as men with severe obesity, Jolles et al. [60] demonstrated that motivations for entering treatment include both physical and clinical goals (weight loss, health improvement, and physical condition) and psychosocial factors (self-esteem and body image), suggesting that treatment initiation is rarely driven by a single trigger.

Regarding barriers to treatment initiation, the literature converges on the relevance of practical and relational obstacles. Anastasiadis et al. [61] discuss how initial participation may be hindered by everyday constraints and contextual aspects of care, including trust, perceived safety, and cultural appropriateness. Similarly, evidence from service-based surveys reported by Look et al. [57] and Salle et al. [56] highlights that gaps in clinical communication and in the way, obesity is addressed during care encounters can lead to postponement, weak patient engagement, and insufficient perception of support to initiate an effective treatment plan.

In the domain of barriers to continuity, studies indicate that treatment discontinuation tends to occur when practical costs accumulate over time (such as time demands and routine constraints), when program models fail to adapt to patients’ life contexts, and when emotional and social factors undermine sustained behavioral change. In this regard, Anderson et al. [62] emphasize that barriers such as a lack of time and financial costs may compromise continuity, while Jolles et al. [60] describe treatment dropout associated with work demands, stress, and difficulties in maintaining regular participation. Complementarily, Miolanne et al. [63] underscore that sustained engagement and maintenance of outcomes depend on continuous support and structured follow-up, suggesting increased vulnerability of treatment trajectories when such support is intermittent.

Finally, regarding motivators for treatment continuity, the literature highlights the combined influence of social support, therapeutic alliance with the care team, and structured follow-up. Gallo et al. [64] einforce the role of the family environment and daily-life conditions in sustaining adherence, while Santos et al. [65] report that patient-centered approaches-characterized by shared decision-making and empathetic communication-promote engagement and long-term maintenance. In addition, Morgan et al. [66] demonstrate that structured digital interventions can support continuity by increasing acceptability, convenience, and self-monitoring, while avoiding overly rigid prescriptions that may undermine adherence. These findings suggest that adjustments in care format, such as follow-up channels, goal setting, and support mechanisms, are as relevant as the prescribed content itself for maintaining patients within the therapeutic pathway. Table 4 synthesizes these criteria and their respective references.

**Table 4:** Barriers and motivators for the treatment of obesity

Aspect	Number	Description	References
Motivations for starting the treatment (Code - C24)	1	Diagnoses of dietary restrictions and/or concerns about physical health	[56–58, 60]
	2	Motivation from family and friends	[56, 60]
	3	Physical, emotional and/or social difficulties	[58, 60]
	4	Medical encouragement	[56, 57]
	5	Negative experiences with weight and/or psychological impacts (social discrimination, bullying, anxiety, depression and low self-esteem)	[58, 60, 65, 67]
	6	Personal motivations (desire to improve self-esteem, well-being and disposition)	[56–58, 60]
Barriers for starting the treatment (Code - C25)	7	Logistical challenges (lack of time, difficulties with transportation)	[58, 59, 61–65, 67–70]
	8	Lack of understanding about treatment options and/or the treatment process	[56, 57, 67, 71]
	9	Shame, stigma and social prejudice	[57, 67, 71, 72]
	10	Financial impact of treatment	[61, 67, 72]
	11	Lack of family and friend support	[63, 71, 73]
	12	Lack of access to health services	[58, 61, 63, 64, 72]
	13	Resistance to lifestyle changes and/or lack of perception of obesity as a health problem	[57, 59, 62, 67]
Barriers to continuing treatment (Code - C26)	14	Seeking quick results	[72]
	15	Overly restrictive diets	[66, 71]
	16	Adverse effects of treatment	[56, 71]
	17	Financial difficulties	[61, 62, 69, 71]
	18	Lack of social and family support and encouragement	[63, 67, 71, 73]
	19	Emotional and psychological problems	[56, 67, 71]
	20	Negative reports about Treatment	[67]
	21	Disappointment with results	[68, 71]
Motivations for remaining in treatment (Code - C27)	22	Improvement in perceived quality of life	[58, 69–73]
	23	Support from support groups and the medical and psychological team	[58, 59, 63, 64, 70–72]
	24	Progress in physical and psychological health	[70, 73]
	25	Regular multidisciplinary follow-up (Support from nutritionists, psychologists, and physical activity professionals improves adherence to treatment)	[56, 62–64, 69–71]
	26	Continuous family and social engagement	[58, 63, 64, 73]
	27	Nutritional and behavioral education (Patients who develop a greater understanding of their eating and behaviors tend to remain in treatment)	[58, 62–64, 66, 69–72]
	28	Personalized diet and physical activity programs	[57, 58, 63, 66, 69]
	29	Setting realistic goals	[56–58, 64, 66, 70]
	30	Initial success in weight loss	[57, 67, 71]
	31	Use of monitoring technologies	[57, 58, 66, 70–72]

### Preliminary FCP-IAOM Framework

**Fig. 3:**
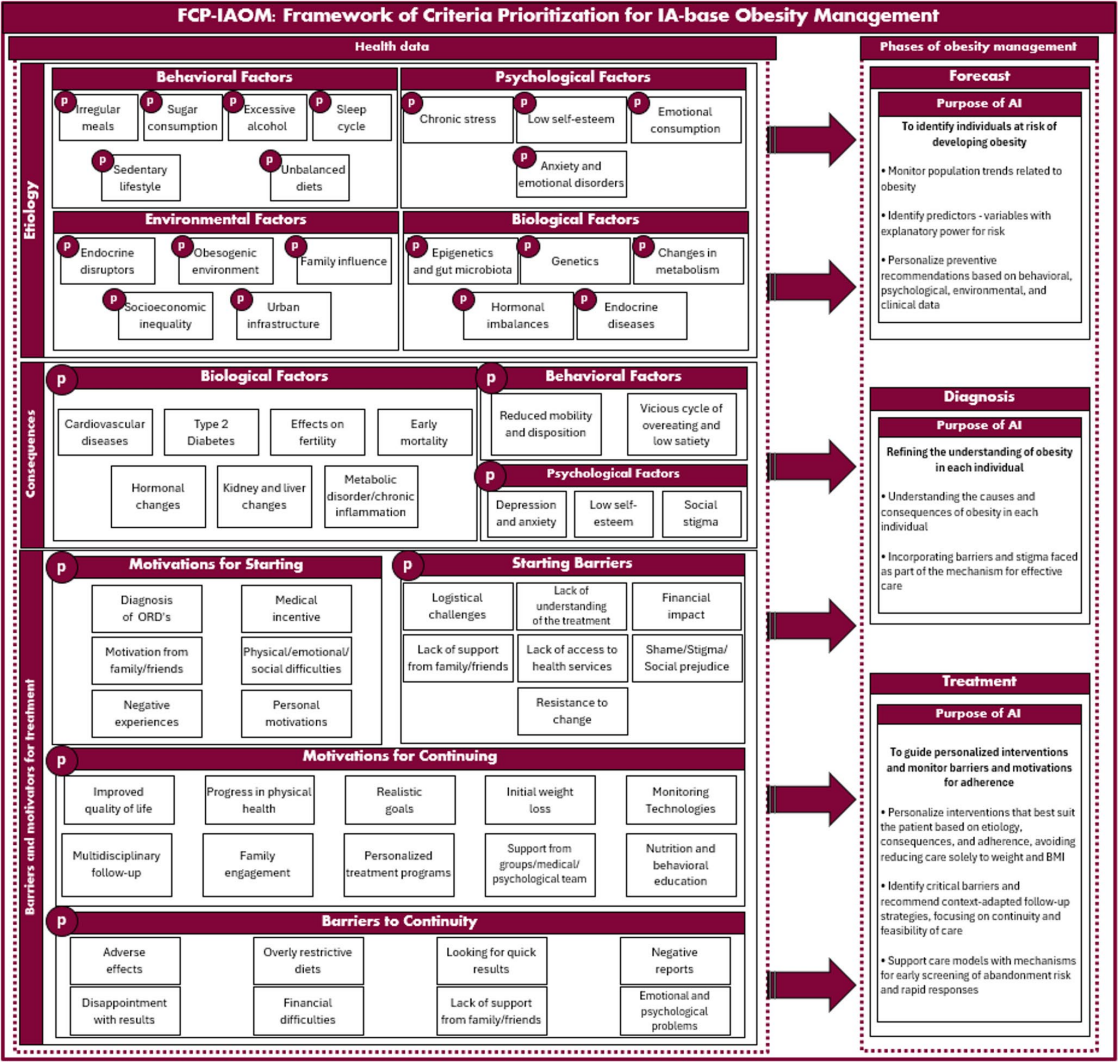
Preliminary FCP-IAOM: framework of criteria prioritization for IA-based obesity management

Based on the synthesis of criteria identified through the SLR and organized into the two thematic axes (I and II), a preliminary systemic framework for obesity management was developed and is illustrated in Fig. 3. Within this framework, health-related data constitute the central focus and are structured into three interconnected layers. The first layer corresponds to obesity etiology, the second to its consequences, and the third to barriers and motivations related to treatment adherence. The etiological layer comprises four multifactorial domains, namely behavioral, psychological, biological, and environmental, whereas the consequences are distributed across three domains: biological, psychological, and behavioral. Treatment adherence–related aspects are organized into four groups: motivation for treatment initiation, initial barriers, motivation for continuity, and reasons for treatment discontinuation. Each domain encompasses a set of specific criteria, as previously described in Tables 2 and 3, enabling obesity to be visualized as a complex system resulting from the interaction of multiple factors rather than a single isolated determinant.

Within the FCP-IAOM, each criterion is accompanied by the letter “*p*”, a symbol used as a marker of the priority value associated with that element. This value is derived from the multicriteria analysis stage through the combined application of the FDM and fuzzy TOPSIS method, which are responsible for consensus, refining, weighting, and ranking the criteria. Accordingly, the framework does not merely list factors related to obesity but organizes them so that each criterion receives a relative weight, reflecting both its clinical importance and its applicability in real-world care contexts.

Obesity management within the FCP-IAOM is structured into three sequential phases, prevention, diagnosis, and treatment, drawing on the logic of the WHO PRET initiative, adapted to a chronic noncommunicable condition. In Fig. 3, these phases are positioned to the right of the health data block and articulate the specific AI objective at each stage, based on the defined criteria. In the prevention phase, the criteria provide a basis for identifying individuals and populations at increased risk of developing obesity. In the diagnostic phase, they support a more in-depth understanding of the underlying causes and consequences in each case. In the treatment phase, they guide personalized interventions and support the monitoring of barriers and motivators related to treatment adherence. Across all phases, AI is presented as a decision-support resource whose effectiveness depends on the quality of the criteria and the previously structured data.

In this way, the proposed framework seeks to move beyond simplistic, reductionist approaches focused solely on body weight, offering a broader, multifactorial perspective on obesity. By integrating behavioral, psychological, biological, and environmental factors, along with clinical consequences and adherence-related dimensions, the framework provides both a conceptual and operational foundation for developing decision-support models that are sensitive to the condition’s complexity and to the diverse perspectives involved in its management.

### Criteria Selection Based on Relevance and Applicability

Based on the relevance and applicability assessments, a selection process was conducted to identify the criteria most suitable for inclusion in the proposed obesity management framework.

The criteria identified in Tables 2 and 3 and synthesized in the proposed framework were evaluated by the expert panel, as described in the Materials and Methods section. Each etiological criterion (C1 to C20) was assessed individually. In contrast, criteria related to consequences and to treatment barriers and motivators were evaluated at the level of aggregated aspects. Within the consequences axis, criteria were organized into three aspects: biological (C21), psychological (C22), and behavioral (C23). For the axis addressing treatment barriers and motivators, the same aggregation logic was applied. Motivation for treatment initiation was evaluated as criterion C24, barriers to initiation as C25, motivation for treatment discontinuation as C26, and motivation for treatment continuity as C27.

In all cases, experts assigned scores using a five-point Likert scale across two dimensions: importance (clinical relevance) and applicability (feasibility of data acquisition in routine clinical practice).

The proposed fuzzy scale was applied to each respondent for each criterion, and the evaluations were subsequently aggregated using the FDM. Next, defuzzification was performed using Eq. (1), yeldeing a single representative value ($\:{G}_{i}$) for both the importance and applicability of each criterion. The mean of the representative values ($\:{G}_{i}$) was then calculated and used as the cut-off threshold $\:\alpha\:$. Criteria representing ($\:{G}_{i}$) values below this threshold in both analyses, relevance and applicability, were excluded and therefore not considered in the subsequent weighting and ranking stage. When a criterion presented a ($\:{G}_{i}$) value below the cut-off $\:\alpha\:$ in only one of the two analyses, it was retained for the next stage but flagged with caution. The resulting defuzzified scores are presented in Table 5.

**Table 5:** Relevance and applicability representative values

Relevance		Applicability	
Criteria	$\:{G}_{i}$	Criteria	$\:{G}_{i}$
C1	6.356	C1	5.231
C2	6.843	C2	5.448
C3	6.787	C3	5.323
C4	6.453	C4	**5.025**
C5	6.731	C5	5.236
C6	6.341	C6	5.282
C7	6.325	C7	5.304
C8	6.512	C8	5.434
C9	**5.603**	C9	5.277
C10	6.722	C10	5.323
C11	**5.485**	C11	**4.465**
C12	**5.485**	C12	**4.786**
C13	**5.491**	C13	5.277
C14	**5.455**	C14	**5.049**
C15	**5.477**	C15	**4.570**
C16	6.731	C16	5.491
C17	6.580	C17	5.440
C18	**5.108**	C18	**4.577**
C19	6.271	C19	5.271
C20	**5.251**	C20	**5.072**
C21	6.777	C21	5.672
C22	6.615	C22	5.383
C23	6.700	C23	5.595
C24	6.713	C24	5.317
C25	6.731	C25	**5.193**
C26	6.632	C26	**5.162**
C27	6.713	C27	5.290
**Cut-off value (α)**	**6.255**	**Cut-off value (α)**	**5.204**

**Fig. 4:**
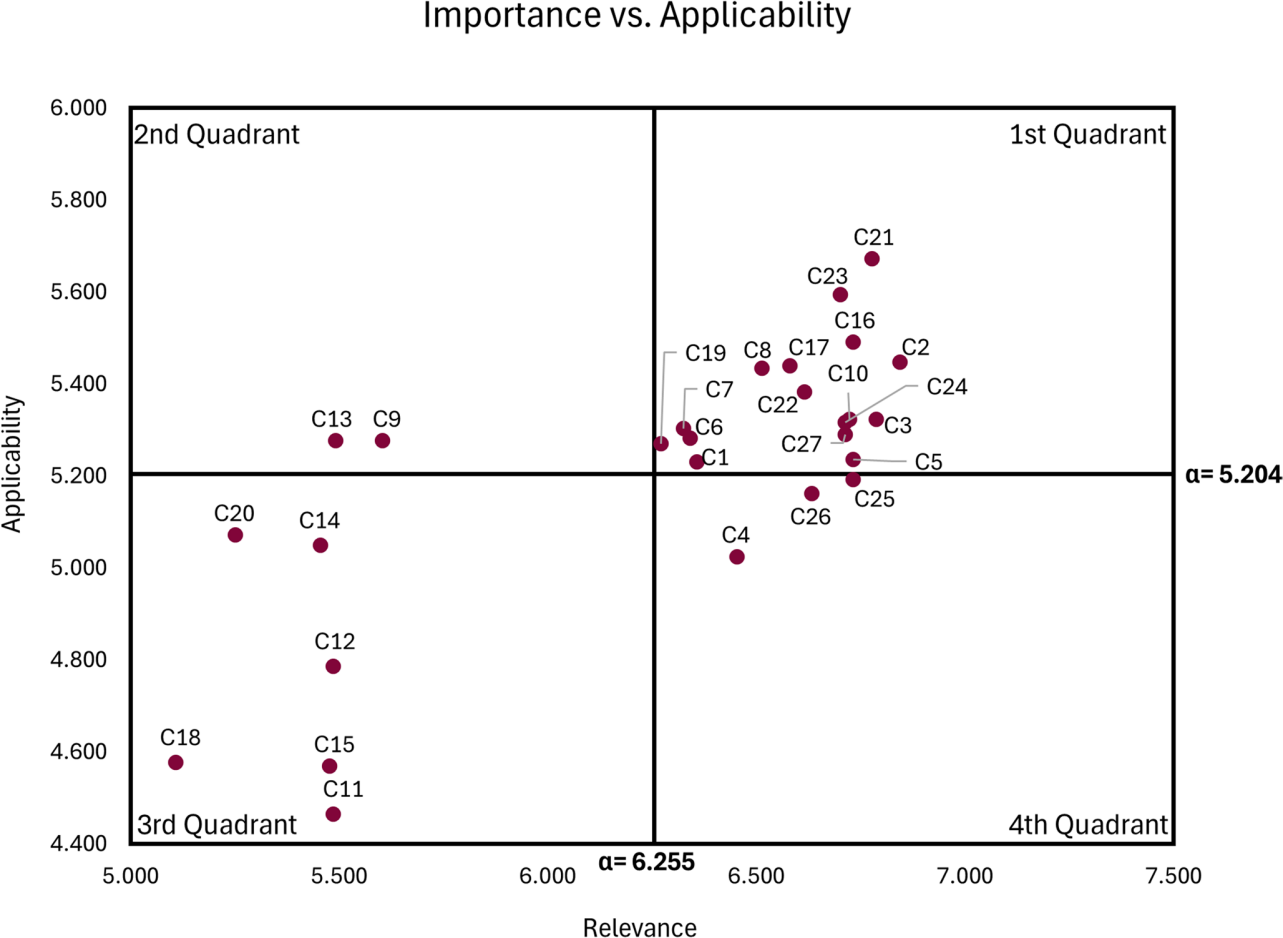
Relevance-versus-applicability diagram

Based on the relevance and applicability results, a quadrant diagram was constructed, positioning the criteria on a two-dimensional plane with importance on the x-axis and applicability on the y-axis. Reference lines were drawn at the respective cut-off values for relevance and applicability. Criteria in the first quadrant, with high relevance and high applicability, were prioritized for use in obesity management because they are both highly relevant and readily accessible, and are therefore recommended to remain in the framework. The second quadrant comprises criteria with lower relevance but high applicability; these are recommended for inclusion in the framework with caution, as their adoption depends on their relevance to the intended objective and the cost–benefit balance. The third quadrant groups criteria with low relevance and low applicability and is therefore recommended for exclusion. Finally, the fourth quadrant includes criteria that are highly relevant but difficult to obtain. These criteria are recommended to remain in the framework with caution and should be monitored, being considered only when feasible data collection strategies are available, such as the use of proxy variables, standardization of records, or integration of multiple data sources. Figure 4 presents the relevance-versus-applicability diagram.

The following criteria were excluded: C11 (genetic predisposition/polymorphisms), C12 (hormonal imbalances), C14 (metabolic alterations), C15 (epigenetics/microbiota), C18 (endocrine disruptors), and C20 (urban infrastructure/school environment/public policies). In general, these criteria were of low relevance and/or low applicability in this study. They typically require complex or costly laboratory assessments, pose challenges for large-scale standardization, have uncertain direct impact on practical prioritization, and/or are redundant with more readily accessible criteria (e.g., C16 obesogenic environment and C19 socioeconomic inequality). In addition, some of their effects can be indirectly inferred from behavioral and environmental variables retained in the framework.

Criteria C4 (excessive alcohol consumption), C9 (low self-esteem), C13 (endocrine diseases), C25 (barriers to treatment initiation), and C26 (motivation for treatment discontinuation) were retained with caution due to an asymmetry between relevance and applicability. These criteria were maintained for the subsequent weighting stage to preserve flexibility in decision-making. Criteria in the second quadrant (lower relevance, high applicability) may be useful when data collection is straightforward and contextually warranted, whereas criteria in the fourth quadrant (high relevance, low applicability) remain relevant but should be considered only when feasible data acquisition strategies are available, such as proxy measures, standardized records, or data integration across sources. This approach enables case-by-case decisions in the subsequent stage without compromising the framework’s parsimony or its focus on accessible and clinically useful data.

### Criteria Prioritization to Support Clinical Decision-making

Following the selection stage, the retained criteria were prioritized to support clinical decision-making in obesity management, with consideration of both their relative importance and practical applicability.

In this context, the selected criteria were submitted to prioritization using the fuzzy TOPSIS method. To construct the fuzzy decision matrix, the same linguistic scale and Saaty discretization (1 to 9) were applied to the relevance and applicability evaluations assigned by the experts. For the fuzzy TOPSIS procedure, expert contributions were weighted according to their level of involvement in obesity management, which was collected using a five-point Likert scale ranging from “no involvement” to “expert in the field.” These responses were also converted into triangular fuzzy numbers (TFNs), as presented in Table 6, forming the fuzzy weight vector used in the subsequent stages of the method. As a result, evaluations provided by experts with greater involvement in obesity management exerted a proportionally greater influence on the calculation of the criteria proximity coefficients.

**Table 6:** Level of professional involvement with obesity management

Level of professional involvement with obesity management	Likert Scales	Fuzzy numbers
No involvement - I do not work with obesity.	1	(1, 1, 1)
Low involvement - I occasionally deal with patients with obesity, but it is not my main focus.	2	(1, 3, 5)
Moderate involvement - I regularly work with patients with obesity, but it is not my main area of ​​practice.	3	(3, 5, 7)
High involvement - I frequently work with patients with obesity and apply specific knowledge in the area.	4	(5, 7, 9)
Specialist in the subject - My professional practice is directly focused on the management of obesity.	5	(7, 9, 9)

The fuzzy TOPSIS method was applied separately to the two evaluated dimensions, criterion relevance and applicability, resulting in two distinct rankings. For application within the proposed framework, the importance and applicability scales were subsequently unified by calculating the arithmetic mean (µ) of the proximity coefficients obtained for each criterion across both dimensions. This mean value was used as a guide for prioritizing data collection and utilization in obesity management contexts. In the context of future AI developments, the proximity coefficient derived from the importance scale will serve as the primary criterion weight (represented by the symbol “*p*” in the framework), whereas the unified ranking will support decisions on which variables to prioritize when data availability or feasibility constraints are present. The results of these rankings are presented in Table 7.

**Table 7:** Criteria priority ranking

Criteria	Relevance		Applicability		Priority	
	$\:{CC}_{i}$	Rank	$\:{CC}_{i}$	Rank	$\:\mu\:$	Rank
C2: Unbalanced diet and/or based on ultra-processed foods, hyperpalatable foods and added food	0.75	**1°**	0.61	**4°**	0.68	**3°**
C3: Excessive consumption of sugar and sugary drinks	0.74	**2°**	0.59	**10°**	0.67	**5°**
C21: Consequences - Biological aspects	0.73	**3°**	0.67	**1°**	0.70	**1°**
C25: Adherence - Barriers to starting treatment	0.73	**4°**	0.55	**19°**	0.64	**12°**
C5: Sedentary lifestyle	0.73	**5°**	0.59	**9°**	0.66	**6°**
C16: Obesogenic environment	0.73	**6°**	0.63	**3°**	0.68	**4°**
C27: Adherence - Motivation to remain in treatment	0.72	**7°**	0.59	**8°**	0.66	**7°**
C10: Emotional eating	0.72	**8°**	0.58	**11°**	0.65	**9°**
C23: Consequences - Behavioral aspects	0.72	**9°**	0.65	**2°**	0.69	**2°**
C24: Adherence - Motivation to start treatment	0.72	**10°**	0.57	**13°**	0.65	**10°**
C22: Consequences - Psychological aspects	0.71	**11°**	0.61	**5°**	0.66	**8°**
C26: Adherence - Motivation to drop out of treatment	0.70	**12°**	0.55	**20°**	0.63	**14°**
C17: Family influence and cultural norms	0.69	**13°**	0.60	**6°**	0.65	**11°**
C4: Excessive alcohol consumption	0.67	**14°**	0.53	**21°**	0.60	**16°**
C8: Anxiety and emotional disorders	0.67	**15°**	0.60	**7°**	0.64	**13°**
C6: Sleep cycle	0.64	**16°**	0.57	**15°**	0.61	**15°**
C7: Chronic and occupational stress affecting eating habits	0.62	**17°**	0.57	**14°**	0.60	**18°**
C1: Irregular meals	0.61	**18°**	0.56	**18°**	0.59	**19°**
C9: Low self-esteem related to body image	0.61	**19°**	0.58	**12°**	0.60	**17°**
C19: Socioeconomic inequality affecting access to healthy food and physical activity	0.60	**20°**	0.56	**17°**	0.58	**20°**
C13: Endocrine diseases	0.57	**21°**	0.56	**16°**	0.57	**21°**

On the relevance scale, criterion C2 presented the highest proximity coefficient, followed by C3 and C21. These results highlight the central role of nutritional factors and the biological consequences of obesity in the expert panel’s prioritization. Criteria related to treatment barriers and motivators, such as C25 and C27, also ranked among the highest, indicating that treatment adherence and continuity are relevant components of obesity management. In contrast, C19 and C13 occupied the lowest positions, suggesting lower relative weights than behavioral, emotional, and nutritional aspects.

On the applicability scale, the prioritization pattern differed slightly. The highest-ranked criteria were C21, C23, and C16, indicating that, in the experts’ view, the clinical and behavioral consequences of obesity are more readily operationalized in practical interventions. Nutritional criteria such as C2 and C3, although highly important, ranked in the middle to lower range of applicability, which may reflect challenges in implementing sustainable dietary changes. Adherence-related factors, including C24, C27, C25, and C26, showed moderate to lower values, suggesting that, although recognized as relevant, they still face practical difficulties in real-world intervention contexts.

Taken together, these results demonstrate that effective obesity management emerges from the interaction between modifiable behavioral determinants, heterogeneous biological profiles, and the practical conditions that sustain treatment adherence over time, providing an empirically grounded basis for the operationalization of the FCP-IAOM framework.

## FCP-IAOM Framework

**Fig. 5:**
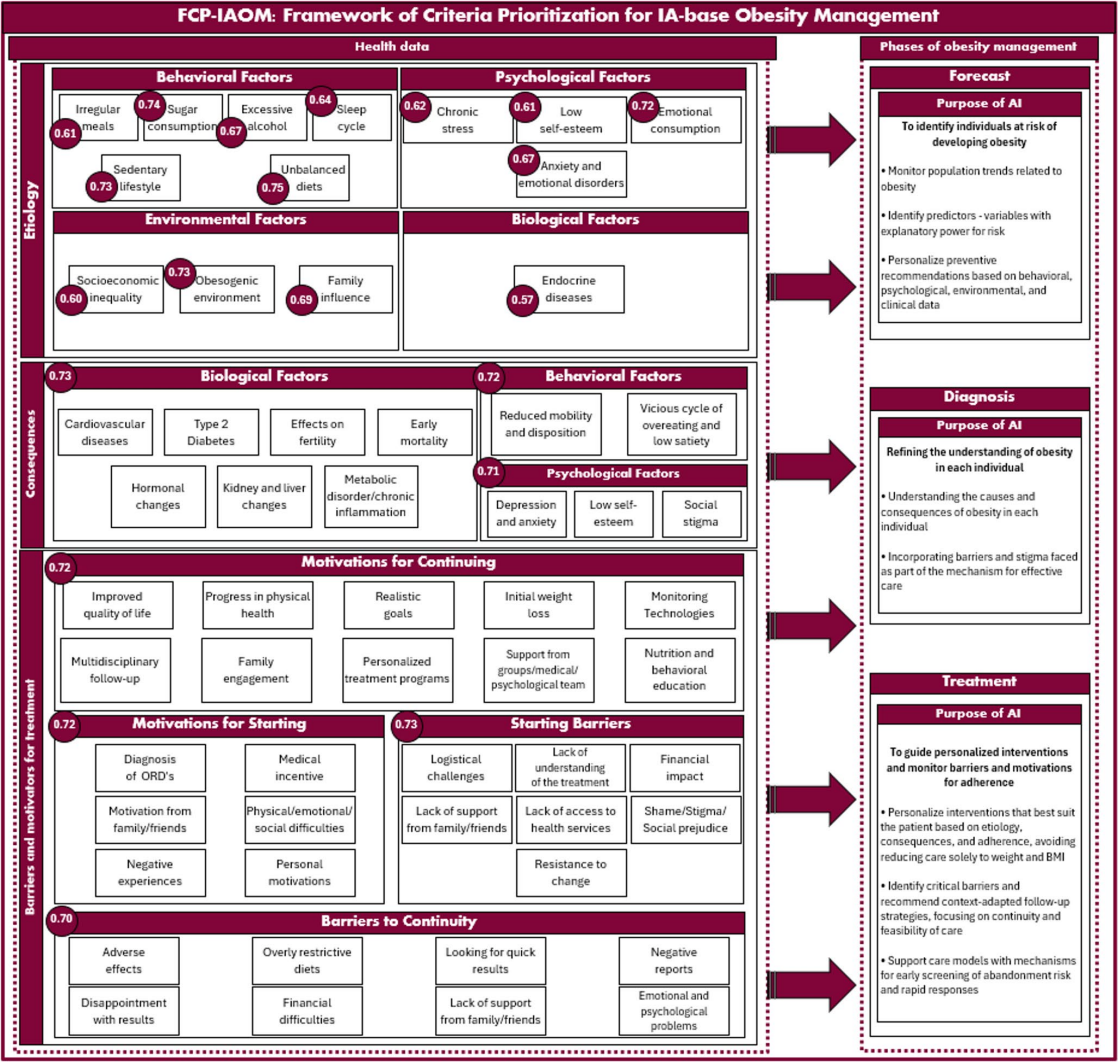
FCP-IAOM framework

This study set out to operationalize the multifactorial nature of obesity by identifying, validating, and prioritizing clinically meaningful criteria for management. The results indicate that effective obesity care depends less on isolated biological mechanisms and more on integrating behavioral determinants, clinical consequences, and the conditions that sustain long-term adherence. In this sens, based on the results, where only the criteria validated by the FDM were retained and the “*p*” values were replaced by the importance scores derived from the application of the fuzzy TOPSIS method, it was possible to propose the FCP-IAOM framework in its final configuration, grounded in expert opinions. Figure 5 presents the FCP-IAOM framework.

The literature shows that although obesity is widely recognized as a chronic condition with complex pathophysiology, care practices remain largely organized around simplified, weight-centered logics. Such approaches tend to individualize obesity and place primary responsibility on the patient [5]. Recent empirical evidence illustrates this disconnect. In the ACTION-FRANCE study, 73.1% of individuals with obesity agreed that weight management was entirely the patient’s responsibility, and participants also reported substantial delays in seeking care and gaps in clinical communication [56]. This pattern aligns with broader sociocultural findings. In qualitative accounts involving adolescents, such as those reported by Gonçalves et al. [42], obesity is frequently associated with attributes such as laziness and personal weakness, reinforcing an individual-centered model in which the person is perceived as the sole determinant of the condition. Similarly, g and Heuer [5] discuss how experiences of weight stigma are associated with poorer psychological outcomes, including depression, and may reinforce maladaptive eating behaviors, such as binge eating, thereby contributing to a cycle that undermines treatment adherence and continuity of care.

At this point, the FCP-IAOM positions itself as an advance over obesity management models that implicitly place responsibility on the individual. Rather than merely reiterating the multifactorial nature of obesity, the proposed framework translates this complexity into a management architecture that makes explicit the connections among the factors that lead to obesity, the consequences that unfold over time, and the conditions that enable or hinder the therapeutic trajectory. Structurally, the framework integrates three interrelated layers, etiology, consequences of obesity, and treatment adherence, and incorporates a dimension often underrepresented in traditional models: the structural and symbolic barriers to care, including stigma and individual blame, which interfere with care seeking, therapeutic alliance, and continuity of treatment. This design choice seeks to move beyond reductionist views that interpret individuals with obesity primarily as lacking self-control, and instead situates obesity within a system of real-world determinants and constraints that must be explicitly operationalized in the design of care. Importantly, the proposed framework is not intended to replace clinical judgment or existing clinical guidelines. Rather, it provides a structured way to organize and prioritize decision-relevant information that clinicians already consider, often implicitly, in obesity management, supporting rather than constraining individualized clinical reasoning.

The phased structure of the FCP-IAOM framework, namely prevention, diagnosis, and treatment, was inspired by and adapted from the WHO’s PRET initiative [8]. In its original formulation, PRET proposes an organizational approach that centers on strengthening reusable capacities and coordinated systems rather than narrowly threat-specific plans. This logic emphasizes equity, coherence, and preparedness through integrated planning across different levels of response [8].

When applied to obesity, a chronic, noncommunicable condition with widespread prevalence and systemic impact, this principle supports organizing care around coordinated, reusable capacities that extend beyond isolated clinical interventions. Accordingly, the adaptation preserves the central PRET rationale that effective management requires more than clinical prescriptions alone, relying instead on integration across levels of care, communication, engagement, and structured routines that enhance the resilience and continuity of obesity care over time [8].

However, because the objective of the FCP-IAOM is to operationalize prioritizable criteria and render them tractable within an AI-supported decision framework, the broader PRET planning cycle was condensed into three sequential phases of care: prevention, diagnosis, and treatment. This simplification does not aim to exhaust the PRET proposal, but rather to isolate the stages that are directly connected to clinical and managerial decisions amenable to computational support. Other dimensions, such as broader intersectoral governance, network architecture, and community mobilization, are therefore preserved as implementation conditions when the model is applied in real-world healthcare settings.

This distinction also facilitates comparison with recent AI-centered models in the literature. Fernandes et al. [74] developed PRIMO, an explainable AI tool that predicts six-month weight loss success from a limited set of early engagement and dietary attributes, supporting early adjustments to intervention strategies. Similarly, Saux et al. [75] proposed the SOPHIA machine learning model to predict five-year BMI trajectories after bariatric surgery, providing an interpretable tool for preoperative decision support. Although both are relevant contributions, they operate primarily within the domain of outcome prediction in specific clinical contexts.

The FCP-IAOM extends beyond this predictive scope by proposing a broader obesity management system. Rather than focusing solely on forecasting outcomes, the framework specifies which patient dimensions should be systematically considered (etiology, consequences, and therapeutic adherence), how these dimensions are organized across phases of care, inspired by the PRET initiative, and why contextual factors, such as barriers and stigma, are integral to effectiveness. In this way, the FCP-IAOM bridges systemic understanding and the operationalization of prioritized criteria, offering a comprehensive foundation for integrating predictive and generative AI modules without reducing the complexity of care. For example, in patients with obesity who repeatedly discontinue treatment, the framework prioritizes the assessment of adherence-related barriers and contextual constraints before intensifying or modifying therapeutic strategies.

The ranking results, following consensus through the FDM and prioritization using fuzzy TOPSIS, indicate that clinical relevance and feasibility of care are not concentrated within a single domain, but rather emerge from a core set of interconnected criteria that integrate modifiable determinants, biological heterogeneity, functional consequences, and real-world conditions that sustain continuity of care. Within the etiological domain, the prominence attributed to behaviors and daily routines, such as maladaptive dietary patterns, sedentary behavior, sleep, and well-being, is consistent with evidence describing obesity as a dynamic system of factors operating over time, in which modifiable risks influence future adiposity trajectories through both direct and indirect behavioral pathways [25]. In parallel, the presence of biological criteria among the most relevant is coherent with literature describing the contribution of genetic mechanisms and neuroendocrine pathways to early-onset phenotypes and individual susceptibility, reinforcing the need to acknowledge metabolic heterogeneity in obesity management [25, 50]. This perspective is further supported by evidence positioning obesity as the product of gene–environment interactions and highlighting the limitations of individualizing approaches when applied in isolation [50]. Within the psychological and behavioral domain, the salience of criteria such as binge eating and dietary patterns characterized by high consumption of ultra-processed foods is supported by studies reporting associations between food addiction and increased obesity risk in adolescents, suggesting that aspects of eating behavior may operate through maintenance mechanisms similar to those observed in chronic conditions with strong biopsychosocial components [44]. With respect to treatment adherence, the ranking reinforces that therapeutic effectiveness depends on the ability of care to be sustained in daily life, rather than solely on the prescription of idealized recommendations. This finding aligns with evidence indicating that, despite recognition of obesity as a chronic condition, clinical practice frequently restricts management strategies to diet and physical activity, while relevant psychosocial impacts, such as anxiety, depression, and social isolation, remain insufficiently addressed, thereby weakening therapeutic alliance, acceptance, and long-term retention in care [56]. Similarly, studies examining access to and attendance in treatment programs demonstrate that adherence is strongly shaped by structural constraints and care-related inequalities, particularly among vulnerable populations [61].

The ranking developed in the present study does more than hierarchize factors; it reveals that the center of gravity of obesity management lies at the interface among relevant behavioral determinants, biological variability, and the psychosocial and structural conditions that make care feasible and sustainable over time.

In this way, the study contributes to consolidating the understanding of obesity as a chronic, multifactorial, and systemic phenomenon by organizing determinants, outcomes, and conditions for continuity within a single operational structure. Rather than treating etiology, consequences, and adherence as parallel domains, the FCP-IAOM makes their interdependencies explicit and provides a prioritized set of criteria, reducing the conceptual fragmentation commonly observed in the literature and creating a consistent basis for comparability across studies and for the development of indicators. Moreover, by linking layers of criteria to phases of care, the framework advances beyond a descriptive level toward an operational one, offering guidance on how this complexity can be addressed longitudinally in obesity care.

## Practical, Clinical, and Organizational Implications

From a practical and clinical perspective, the proposed framework and prioritized criteria indicate that more effective obesity interventions depend not only on defining clinical targets but also on early identification of key enablers and barriers to care. These include time constraints, daily routines, costs, social support, emotional distress, stigma, therapeutic alliance with the healthcare team, and the adequacy of the program format. In practice, this perspective favors a more realistic approach to obesity management, in which therapeutic plans can be adjusted according to patients’ capacity to implement recommendations in daily life, incorporating gradual goals and continuous monitoring of factors that commonly precipitate treatment discontinuation.

At the service and team levels, the findings indicate a need for greater flexibility in care delivery, including digital channels, telemonitoring, self-monitoring tools, asynchronous follow-up, and re-engagement strategies. Importantly, these elements should not be treated as ancillary technological add-ons but as structural components of care models designed to reduce friction and support long-term continuity.

At the organizational level, the study highlights that treatment dropout and low adherence should not be interpreted primarily as individual failures but as indicators of misalignment between service design and the lived realities of people receiving care. A direct implication of this work is the need to orient care models toward strengthening continuity through clearer care pathways, multiprofessional integration, regular points of contact, early identification of dropout risk, and timely responses. In addition, the framework provides a basis for defining quality-of-care indicators that go beyond body weight and BMI, incorporating dimensions of functioning, well-being, adherence, and barriers to care, thereby supporting improvements in service management, auditing, and local planning.

From a broader social perspective, the study shifts the focus from individual behavior to conditions of care and reinforces the role of social determinants in obesity management. By explicitly incorporating stigma, responsibility attribution, and structural barriers as criteria, the framework supports the view that effective strategies require intersectoral action, including anti-stigma public and institutional communication, access to food and physical activity resources compatible with local contexts, reduction of economic barriers, and strengthening of social support networks. In practical terms, this implies that policies and programs should be designed with sensitivity to inequalities, avoiding intervention models that, while technically sound, are unattainable for segments of the population.

Finally, as a cross-cutting implication, the FCP-IAOM serves as a map of variables oriented toward the longitudinal management of obesity. It supports the design of intelligent systems that go beyond risk prediction alone, sustain clinical decisions, signal critical barriers, and recommend context-adapted follow-up strategies, with a focus on feasibility, continuity of care, and person-centered approaches.

## Conclusion, Limitations, and Future Research

This study aimed to identify, organize, and prioritize criteria related to the etiology, consequences, and barriers and motivators for obesity treatment. The process produced a validated set of criteria, hierarchically structured by relevance and applicability, and a systemic framework that conceptualizes obesity across multiple interconnected dimensions. For endocrinologists and multidisciplinary teams involved in obesity care, the FCP-IAOM provides a structured framework for integrating clinical judgment, patient context, and emerging digital tools while adhering to established standards of care.

Taken together, these findings provide a conceptual and operational basis for the multifactorial management of obesity, contributing to a shift away from simplistic views that focus solely on body weight or isolated determinants. The proposed framework may support the planning of clinical and public health interventions and the future development of decision-support models, including Artificial Intelligence applications that use the prioritized criteria as inputs. In this way, the study advances toward a more integrated approach to obesity management, one that is sensitive to the condition’s complexity and aligned with the real-world needs of individuals and healthcare services. Additionally, the resulting framework should be understood as an intermediate step between a systemic understanding of obesity and its computational implementation. Because it has not yet been validated in real-world care settings, inferences about performance, acceptability, and effectiveness when operationalized within healthcare services remain limited.

At the same time, several limitations should be acknowledged. Although the SLR and the expert elicitation process yielded a structured and validated set of criteria, some criteria still reflect a tension between relevance and applicability. This reflects practical constraints related to data collection and standardization, particularly when a criterion is considered clinically important yet has limited operational feasibility in clinical and service-based contexts. This asymmetry was deliberately incorporated into the framework by retaining certain criteria with caution, preserving flexibility and enabling case-by-case decisions in subsequent stages without compromising the model’s parsimony or its focus on accessible and clinically useful data. In addition, during the expert evaluation stage, criteria related to consequences and to treatment barriers and motivators were necessarily assessed at the level of aggregated dimensions rather than individually, unlike the etiological criteria. This approach may reduce analytical granularity and limit direct comparisons of priority among subcomponents within these dimensions.

Finally, the proposed framework should be understood as an intermediate step between a systemic understanding of obesity and its computational implementation. Future research should advance the operational translation of the prioritized criteria into measurable variables by developing metrics, protocols, and data collection instruments for clinical, behavioral, psychosocial, and contextual data, recognizing that a single criterion may require multiple indicators and data sources. Further work is also needed to specify how the framework can be coupled with decision-support models and AI-based solutions, including the selection of appropriate techniques, models, and architectures for each phase of obesity management. The next step is to evaluate the framework in real-world care settings, assessing predictive accuracy, adherence-related outcomes, continuity of care, equity, operational feasibility, and clinical utility, thereby consolidating its role as a bridge between prioritized criteria and intelligent, person-centered decision-support tools.

## Data Availability

The data will be made available on request.
